# A cash lottery increases voter turnout

**DOI:** 10.1371/journal.pone.0268640

**Published:** 2022-06-03

**Authors:** Raymond J. La Raja, Brian F. Schaffner

**Affiliations:** 1 Department of Political Science, University of Massachusetts Amherst, Amherst, Massachusetts, United States of America; 2 Department of Political Science, Tufts University, Medford, Massachusetts, United States of America; Georgia State University, UNITED STATES

## Abstract

Reform efforts to improve turnout typically focus on reducing the costs of participation or strengthening appeals to civic duty. While these efforts generate modest effects, this paper explores whether citizens might respond to extrinsic rewards to encourage voting. We conduct a field experiment offering lottery prizes to undergraduate students in conjunction with a student government election at a major public university. We find that extrinsic rewards appear to boost voting significantly in these low turnout elections and that the effects of a lottery appear to be especially strong among those of lower socio-economic status.

## Introduction

The search for solutions to low voter turnout typically emphasizes minimizing the costs of voting. These strategies include making voter registration automatic, increasing access to mail-in ballots, and early voting. The research on the effects of these cost-reducing reforms show that such policies typically have small effects on turnout and generally make voting easier for those who would already participate [1, 2]. Some recent work, however, has begun to explore whether increasing the benefits of voting might bring more citizens to the polls. Most of these studies focus on the psychic value of fulfilling citizen duties [3, 4] and social norms surrounding civic participation [5].

We take a different approach by considering the potential impact of tangible, selective benefits on turnout. A handful of studies have begun to look at whether selective benefits, in the form of cash payments, might increase participation [6–8]. Our analysis differs from these previous studies by conducting an experiment that randomly assigns whether voters are entered into a lottery for a chance at winning a relatively significant sum of money if they choose to vote. Previous experimental studies, in contrast, offer to defray expenses for voting in small (guaranteed) amounts that range from $2-$25 [7, 8]. We believe lottery games are more likely to attract attention and engage voters without additional cognitive or time costs associated with previous experiments, which required filling out surveys or forms. We thus use a low-cost lottery-based approach. For example, a study in London found that a lottery increased the number of residents who registered to vote in a city borough election [6]. Our experiment builds on this research in several ways. First, we use a university election to understand how these findings generalize to low turnout elections in the United States. Second, we examine differences in race/ethnicity and socio-economic status to understand the potential for closing the participation gap between high and low-propensity voters.

As the first study in the U.S. to use a randomized lottery to incentivize turnout, our experimental focus is on a college student population. Given that young people in the U.S. vote at rates lower than any other segment of the electorate [9], understanding how to incentivize this group is a high priority of many mobilization campaigns [10]. While future work should consider how the findings generalize to other contexts, it is important to acknowledge that it is not currently possible to run a lottery-based experiment in most state and all federal elections given electoral regulations and restrictions. Therefore, as the first test of how lotteries incentivize American voters, a college student sample provides novel insight into how these reforms, or similar incentives, might be leveraged in the future.

That said, we believe the findings from a student election have broader applicability, particularly for lower salience elections. Student elections are notoriously low turnout events, with an electorate that varies considerably in political interest and engagement [11], and is challenging to mobilize [12]. The findings here have implications for other low turnout elections, such as those for subnational offices. Additionally, our analysis of treatment effects across groups that vary in political engagement provides insight about the kind of mobilization strategies that might engage low-propensity voters [2]. Finally, the use of lotteries has potential wider applications to stimulate participation in positive forms of collective action beyond elections. In the United States, for example, state and local leaders experimented recently with offering lotteries to raise the rate of vaccinations in their communities. While the efficacy of these efforts is not yet clear, from a policy perspective it would be helpful to understand the types of incentive interventions that might work, and under what conditions, to increase mass participation in socially desirable activities like voting and being vaccinated [13].

## Selective incentives and voter turnout

Widespread voting in democracies can be conceived as a public good. Higher turnout in elections should, in theory, produce positive societal benefits including better representation of the eligible electorate, broader responsiveness and enhanced government legitimacy. And yet the median turnout in recent national elections has been 68% of registered voters in OECD nations [14]. Moreover, turnout is especially low in subnational elections. One analysis of 144 US cities over time estimated an average turnout of 26% of the voting age population [15]. Polities have tried to address the challenge of weak turnout through several means, especially through education and socialization to induce a sense of civic duty about voting. Additionally, there have been efforts to lower the marginal cost of voting in many countries, by making registration easier and providing vote by mail and earlier voting. These have had some small effects [1], and most appear to increase the participation gap between low and high-propensity voters [2].

From a theoretical perspective voting reflects a collective action problem for democracies. Powerful solutions to such problems involve either coercion, such as compulsory voting [16], or selective incentives to induce participation [17]. Many democracies have tried the former approach by requiring citizens to vote, which has boosted turnout [18]. As for the second option, providing individual benefits to induce voting is typically illegal, although in practice political parties in many nations engage in vote-buying, which is a major focus of study in developing democracies [19–21]. The challenge of boosting turnout is especially difficult among voters from marginalized groups who often have fewer resources and civic skills [22], who may not be socialized as much in norms of civic duty [3, 5, 23–26], and who belong to social networks where voting is not prioritized [27, 28]. Recent events related to vaccinating populations against COVID-19 illustrate some of the same problems with eliciting participation from marginalized groups.

We theorize that offering selective benefits potentially alters the behavior of citizens by increasing the incentives for voting, as well as potentially generating patterns of social influence that might engage them to participate. Regarding the stakes, a selective incentive may attract the attention of members of the electorate who are weakly interested in politics, face higher cognitive costs, or who feel an election is not that important. For some citizens the perceived potential to get a material benefit overcomes the cost of voting and lack of interest. Moreover, offering selective benefits might have a differential impact on lower propensity voters. A subset of citizens–often from marginalized groups–may feel less social pressure to turnout, or hold exclusionary beliefs about who should participate, and are more willing to defer to others who they believe know and care more about an election [29]. Overall, the potential to accrue a material gain could provide the nudge that overcomes apathy or a sense that voting is for those who are better informed or who care more about the outcome.

There is at least a second possible mechanism by which lotteries might induce greater voting, although we cannot evaluate it given our limited data. Lotteries plausibly engage a different set of social networks, which are not typically attuned to elections or wedded to norms of civic duty. For example, those with lower socio-economic status are less likely to be part of networks where individuals discuss politics, support norms about participation and mutually-mobilize one another through shaming or encouragement [25, 27, 28]. Studies show that lotteries attract disproportionately players from lower socioeconomic status groups [30] and that frequent players enjoy the excitement and social discussions surrounding the game of chance [31]. Thus, a lottery potentially stimulates networks of citizens who are not typically engaged politically.

For this reason, we might expect a lottery to have a larger impact on subsets of voters who are infrequent voters, particularly in low information campaigns. While this particular study does not directly test the secondary impact of social influence (since the lottery treatment was targeted to specific students), our findings do provide evidence that lotteries may mobilize lower propensity voters.

Despite widespread prohibitions in the U.S. on using selective incentives to stimulate turnout, there have been some important efforts to understand its impact (while staying within the federal and state laws). In one well-designed study in California, residents of two towns were randomly assigned to receive a non-partisan mailing that promised financial compensation of $2, $10 or $25 for voting [7]. The results from this experiment were mixed. However, the study showed a positive yet small boost in turnout. For each additional $10 offered, an individual’s probability of voting increased by 1.5 percentage points. Thus, the lesson from this work appears to be that relatively large financial rewards may be needed to generate significant increases in voting.

Building on this research, we hypothesize that a generic lottery will attract additional voters to the polls by increasing the perceived probability of receiving a benefit for voting. Recent lotteries in two US cities appeared to increase turnout according to commentary, although to the best of our knowledge there are no studies that have evaluated the underlying causes of the increase [32]. To avoid running afoul of the law, our experiment was implemented in conjunction with student government elections on the campus of a major public university in the United States. A previous study of a lottery game focused on efforts to register voters in a London borough, finding an increase of just under 2 percentage points from a baseline of roughly 46% [6]. Our study focuses on a voting population that has been found to be very challenging to mobilize in the context of student elections [11, 12].

Unlike previous experiments we are able to use data on race/ethnicity and first generation status collected by the university to examine whether the treatment effects vary across key demographic groups. The university defines first-generation as students who do not have a parent who has attained a bachelor’s degree. We also collected data on gender but did not anticipate differential voting rates. The findings are reported in S2 Table. First-generation students tend to come from families of lower socioeconomic status, as measured by parental education and income. Moreover, we expect first generation students to be less socialized about the college experience, or integrated into social networks that encourage participation in non-classroom activities like student elections.

White Americans and individuals with higher socio-economic status tend to vote at higher rates than their counterparts because they generally face lower information costs and tend to be embedded in social networks with stronger civic norms [3, 5, 25, 27, 28, 33]. The selective incentive of a lottery may be especially influential in mobilizing groups who are otherwise at a disadvantage when it comes to these civic resources. Thus, we expect the effects of a lottery will be more powerful among more marginalized groups such as people of color and lower SES individuals (measured in this study by first generation status).

## Methods

We conducted a field experiment at the University of Massachusetts Amherst, a large flagship state university in the United States. In February 2019, the university’s Student Government Association (SGA) held its annual elections for president, vice president, and a number of other offices. All undergraduate students are eligible to vote in these elections and voting happens online through a secure website over a three-day period. In the previous year (2018), 17.8 percent of students voted, a turnout rate that is similar to the median rate for mayoral elections in the largest American cities [34]. Our experiment involved randomly assigning a subset of students into a condition where they would be entered into a lottery to win a cash prize if they voted. The study was approved by the University of Massachusetts Amherst Institutional Review Board [35]. Owing to the nature of the study and the fact that subjects were only being asked to vote, subject consent was waived for this research.

All 24,829 active undergraduate students were eligible to participate in the SGA elections. In January we received a list of current students who we randomly assigned to either a control group or one of two treatment groups. The list we received was generated several weeks before the beginning of the semester, so some of the students on our list were no longer active students when the election occurred, while other students had enrolled since we generated our list. Our analysis only includes students who were on the list that we randomized–a total of 22,680 undergraduate students. In the S1 Table we show that the control and treatment groups were well balanced on race/ethnicity, sex, and first-generation status.

First, 3,779 eligible students were assigned to a treatment condition where they received an email from a professor in the political science department at their university (one of the authors) reminding them about the election and informing them that if they did vote they would be entered into a lottery where they would have 5 chances to win a $300 prize. We call this the lottery email. Students were highly unlikely to have had this professor as an instructor, and the email was a generic call to civic participation emanating from the professor’s scholarly interests (see full text below). After the election, we randomly selected five students from among this group who had a record of voting in the election.

In order to isolate the effect of the lottery from the effect of simply being contacted by the professor, we also included a second treatment group of 3,778 eligible students who also received an email, but without the lottery entry. Specifically, students in this group received an email from the same professor at their university reminding them about the student government election and encouraging them to vote. We refer to this as the encouragement email. This email was identical to the email received by the lottery treatment group, except that information about the lottery was removed. The full text of the email is presented below (with the treatment text in brackets):

Elections are being held this week, Feb 19–21, for representatives to the Student Government Association. As a professor who studies elections, I urge you to vote to choose your leaders, make your voice heard, and participate in the civic life of the [REDACTED] community! Last year only 16% of students voted. [***You have been picked randomly from just 1 in 5 students to participate in a raffle for a chance to win $300 IF YOU VOTE*.** ***Five*** ***voters will be selected at random and each will win $300***. You will only be eligible for this reward if you vote. The winnings will be donated through my account at the political science department.]
You will receive emails and/or social media providing you with information about candidates and indicating the links where you can vote at the campus site. I encourage you to inform yourself and VOTE!

To minimize our intervention in the election, most students were assigned to the true control condition where they received no email at all. The size of the two treatment groups was then calculated based on a power analysis which determined the minimum group sizes needed to detect a treatment effect of at least 3 percentage points between the encouragement email and the lottery email conditions while assuming a significant amount of non-compliance. Students in each of the treatment groups received an initial email the day before the start of the election period and then a reminder email on the last day before voting closed.

The email was sent via an application that allowed us to track whether it was opened by the student. Overall, about 70% of the emails were opened by students who received them and students in the encouragement email condition were actually more likely (72.2%) to have opened the email than those in the lottery email condition (68.4%). The difference in open rates is significant (p < .001). Importantly, however, a student would not need to open the email to be treated. The encouragement email had a subject line that read “Vote in SGA elections” and the lottery email’s subject line was “Vote in SGA elections for a chance to win $300.” Thus, there was sufficient information included in the subject line of each email to treat even students who did not subsequently open the emails. For this reason we assume that all students assigned to the treatment groups did receive the treatment, though we also calculate treatment effects only for those who opened the emails.

Students had to navigate to a designated campus webpage to vote, enter their identification information, and then pick from lists of candidates running for student government. The email we sent them deliberately avoided instructions on how to vote and did not provide a link to the voting webpage to ensure that students still faced the same opportunity and cognitive costs in choosing to vote regardless of whether they received the email.

Because SGA was concerned about privacy, the individual-level turnout data was not released to us. Instead, we worked with the University’s Technology Services department to provide them with lists of student IDs depending on the condition to which students were assigned and whether they had opened the emails we sent them. The Technology Services department then returned raw counts of voters and non-voters for each group to allow us to calculate the treatment effects. They also provided us with these figures broken down by three traits: sex, race/ethnicity, and whether the student was a first-generation undergraduate. We use these figures to explore heterogeneous treatment effects. However, since we did not receive individual-level data we cannot estimate regression models on the data and rely instead on difference of proportions tests. Among those in the lottery condition who voted, Technology Services randomly selected five students and provided their names and emails to make the prize award.

### Potential considerations

One concern is that students may have doubted that the lottery was real; after all, it is unusual to be entered into a lottery in exchange for voting. We received one email from a student in the lottery condition inquiring about whether the email was legitimate. It is possible that many other students doubted its authenticity. Indeed, as noted above, the lottery email was less likely to be opened by students than the encouragement email. If there were doubts among some of those receiving the email, then this would lead to a downward bias in the size of the treatment effects we detect.

A second potential concern is whether the email introduced concerns in the minds of students about whether their vote was private. We did not receive any emails from students voicing such concerns. Nevertheless, if the email did prime students to worry about their privacy, then this also may have led to a downward bias in the treatment effects of receiving the lottery email.

A third potential concern relates to the fact that the email came from a professor (one of the co-authors) and invoked language related to social norms about voting. Even though we conducted the experiment at a very large university, it is true that this email may have been especially influential since some students may have been familiar with the professor who sent it or may have paid attention to the email simply because it came from a faculty member. This is similar to how lotteries may actually be advertised in real life; indeed, Covid-19 vaccination lotteries have been messaged by governors in states like Ohio and Massachusetts. In any event, the key design feature of our experiment is that we randomly assigned two different versions of the email, allowing us to compare behavior among those who simply received the encouragement email to those who received an email about the lottery. This allows us to isolate the effect of being in a lottery separate from receiving an email about the election, regardless of who sent the message or the invocation of social norms about voting.

Finally, because campuses are home to tight and overlapping social networks, there may be concerns about spillover effects from our experiment. For example, students in the lottery condition may have told others who were not assigned to that condition about the lottery. This may have had one of two effects. On one hand, students not in the lottery condition may have also become more motivated to vote under the assumption that they were also eligible for the lottery. This would have the effect of biasing our treatment effects downward. On the other hand, if students who heard about the lottery understood that they were not eligible for it, it may have upset them, leading them to participate at a lower rate than they would have otherwise. This would effectively inflate our treatment effects by reducing turnout among the control group. While we cannot be certain that this did not happen, we note that we did not receive any emails from students not assigned to the lottery treatment inquiring about their eligibility for the lottery or expressing dissatisfaction with not having been part of the contest.

## Results

The overall turnout rate in the 2019 SGA election was 15%, down slightly from participation in 2018. Among students on the list we randomized in January, turnout was 17.25%. The rate for our list is higher because it excludes approximately 2,000 students who matriculated at the beginning of the semester and were therefore less likely to know about and participate in student government elections.

Table 1 shows that turnout varied significantly across our experimental groups. Among students who were assigned to receive no email, just 15.90% voted. In the group receiving the encouragement email (without the lottery entry), turnout was 17.97%, and in the group receiving an email with the lottery, 21.91% of students voted. Table 1 shows treatment effects for each condition. The table shows results for two treatment effect calculations: (1) the intent-to-treat (ITT) effect comparing turnout among all students assigned to each condition; and (2) the treatment effects on the treated (ATT) comparing turnout among students in each treatment group who opened at least one of the emails they were sent. For the ATT calculation, we isolate individuals who opened the email under the assumption that those who did not open an email were unlikely to have seen the information about the lottery.

**Table 1:** Treatment effect calculations for encouragement and lottery email treatments.

Comparison groups		ITT
No email	Encouragement email	
.1591(N = 15,123)	.1797 (N = 3,778)	.0207 (p = .002)
No email	Lottery email	
.1591 (N = 15,123)	.2191 (N = 3,779)	.0601 (p<.001)
Encouragement email	Lottery email	
.1797 (N = 3,778)	.2191 (N = 3,779)	.0394 (p<.001)
Among those opening email		ATT
Encouragement email	Lottery email	
.2030 (N = 2,729)	.2677 (N = 2,585)	.0647 p<.001)

ITT p-values based on a difference of proportions test using intent-to-treat calculations. ATT p-value based on a difference of proportions test among those opening the treatment emails.

The first row of results in the table compares turnout among those who received the encouragement email to those who did not receive any email. The first treatment effect shows that simply receiving an encouragement email increased turnout by 2 percentage points (p = .002). However, the increase in turnout was three times as large for students who received the email telling them that voting would enter them into a lottery (ATE = .0601, p < .001).

While comparing participation to the no email group is instructive, in order to isolate the effect of the lottery (separate from receiving an email about the election), we focus on comparing turnout in the encouragement email group (those receiving an email without the lottery) to those in the lottery email group (those receiving the email with the lottery information). Here we observe an increase in turnout in the lottery group that is fairly large in magnitude and statistically significant at p < .001. Students in the lottery condition were 3.94 percentage points more likely to vote than those in the encouragement email group. The 95 percent confidence interval for this increase in turnout ranges from 2.1 to 5.7 points. The estimated effect of 3.94 percentage points amounts to more than a 20 percent increase over the baseline turnout when students only received an encouragement email and nearly a 25 percent increase over the turnout rate in the group that did not receive an email at all. The magnitude of this effect is quite large.

When comparing turnout rates only among students who opened at least one email, we find a 6.47 percentage point increase in the lottery condition over those in the encouragement only condition. The 95 percent confidence interval for this increase ranges from 4.2 to 8.7 percentage points. The estimated 6.47 percentage point increase in turnout amounts to more than a 30 percent increase in participation over the turnout rate in the encouragement email group.

We now turn to comparing treatment effects across subgroups. Student government elections at the university where our study is centered tend to see similar turnout gaps as exist for other elections in the United States. For example, in our pure control group (the students who received no email), there was a 3 percentage-point gap in turnout between white students (18.2% turnout rate) and students of color (15.2% turnout rate). This gap was statistically significant at p < .001 and is consistent with the racial gap in turnout that persists in American elections [36]. We also find in our control group that students who were the first in their family to attend college had a turnout rate that was nearly 5 percentage-points lower than those who were not first-generation college students (18.4% to 22.7%, p < .001). This finding is consistent with research on engagement in campus activities of first-generation college students [37]. Similarly, in the 2020 presidential elections, first generation American citizens had a turnout rate that was nearly 7 percentage-points lower than that for native-born citizens [38]. Thus, at least on these two dimensions, the inequalities in participation in national elections do appear to reproduce themselves even in student government elections.

The university was able to provide us with data on the race/ethnicity of students as well as whether the student identified as a first-generation to attend a university on the CommonApp. The relatively small number of students in most racial/ethnic groups leads us to simplify the race/ethnicity comparison to (non-Hispanic) white versus non-white students. However, in S2 Table we break out the effects for some of the more populated racial/ethnic subgroups. Of particular interest for us is the comparison of first-generation undergraduates to those who come from university-educated households (undergraduate level). Since these first-generation students tend to come from households with lower socio-economic status, they may be less likely to have been socialized to see voting as a duty or embedded in social networks that would increase social pressure or political interest in voting. Indeed, among the true control group (students who did not receive either an encouragement email or the lottery email) turnout among first-generation students was just 18%, compared to 23% among students who were not first-generation. These students are also more likely to come from households with less financial security, meaning that they may be more sensitive to financial incentives.

**Fig 1:**
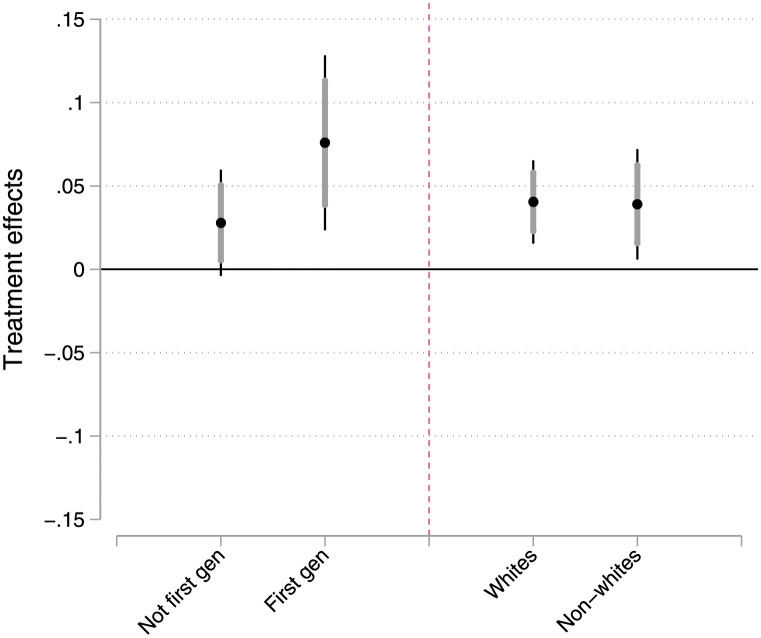
Treatment effects by first-generation status and race/ethnicity. Plot shows point estimates for difference of proportions tests comparing students in lottery email condition to those in encouragement email condition. Thin vertical lines are 95% confidence intervals while thick vertical lines are 84% confidence intervals. If 84% confidence intervals do not overlap, we can be 95% confident that the two estimates are different.

Fig 1 plots the treatment effects for each of these groups. First, the treatment effects appear similar across race/ethnicity. The treatment effect for white students is 4.0 percentage points compared to 3.9 percentage points for non-white students. We see less similarity, however, when we compare the treatment effects based on first-generation status. First generation students in the lottery condition were 7.6 percentage points more likely to vote than first generation students in the encouragement email condition (p = .004). By comparison, the treatment effect for non-first-generation students was less than half that size, 2.8 percentage points (p = .081).

While this difference is suggestive that first-generation students were more influenced by the lottery, the small number of first-generation students (N = 1,026) means that the confidence intervals for those treatment effects are quite large. Thus, we cannot be 95% confident that the treatment effect for first-generation students is larger than that for non-first-generation students.

**Fig 2:**
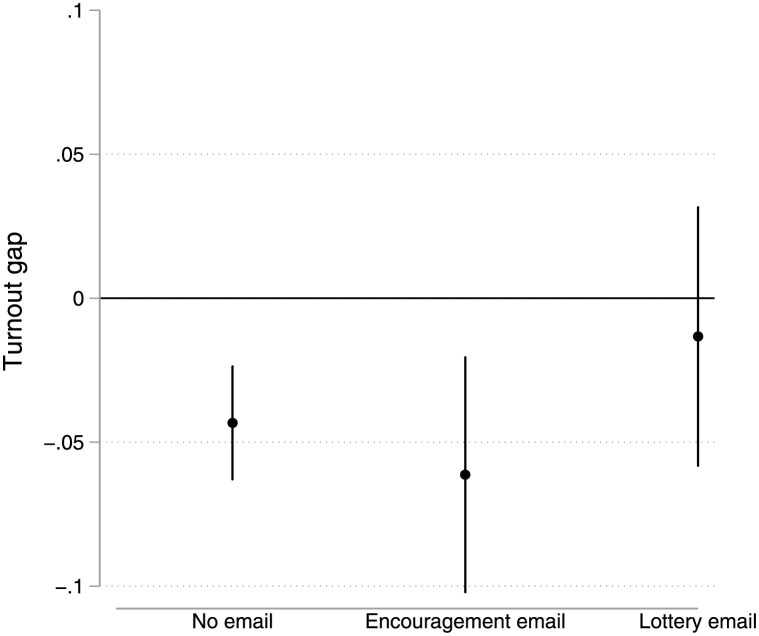
The turnout gap between non-first-generation students and first-generation students in each condition. Plot shows difference in proportion of non-first-generation and first-generation students voting in election. Vertical lines are 95% confidence intervals.

Fig 2 provides an alternative way of looking at the potential for a lottery to make turnout more equitable. Here, we show the turnout gap between non-first-generation students and first-generation students in each of the three randomly assigned groups–those not receiving an email, those receiving an encouragement email, and those receiving the lottery email. In the no email and encouragement email conditions, first-generation turnout is significantly lower than that of non-first-generation students (by roughly five percentage points; p < .001 and p = .005, respectively). However, in the lottery email condition, this gap mostly disappears, with just a 1 percentage point (non-statistically significant, p = .564) difference in turnout. The attenuation of the participation gap is noteworthy because many popular electoral reforms actually appear to *increase* the participation gap between high and low propensity voters [2].

## Conclusions

We have attempted to understand whether tangible incentives can lead to increased and potentially more equitable turnout. Our field experiment shows that voters can be motivated by a simple lottery to participate in elections. Students who received the email offering the lottery were roughly 4 percentage points more likely to vote than those who were simply encouraged to vote. This reflects a 20 percent increase over baseline turnout. Given that the expected payout was well under $1, these treatment effects are impressive. Of course, an encouragement email is costless while our lottery required a $1,500 investment. If we apply our treatment effects to the full student body population we can estimate the cost of each additional individual mobilized by a lottery. Specifically, turnout among the encouragement group was 17.97%, which would produce 4,326 voters if applied to the full eligible voter population at the university. By comparison, the 21.91% turnout rate from the lottery group would correspond to 5,275 voters if the lottery had been applied to everyone. This means that the lottery would produce 949 more voters than just the encouragement email, a cost of $1.58 for each additional voter. If scalable and generalizable to other settings, this would be an efficient way to generate additional voters compared to other common mobilization practices which typically cost much more [39].

Two important caveats are in order regarding broader generalizations about the findings. First, we observed a low salience, student government election in which 15–20% of those eligible typically vote. We cannot be sure that we would realize the same gains in higher turnout elections with elevated stakes and which receive considerable media attention. Nonetheless, even if the dynamic applies mostly to low salience elections, the implications are important for turnout in municipal and other low-turnout contests. Moreover, we observe similar patterns of participation across subgroups in student elections as in U.S. local, state and federal elections. That is to say, subgroups in the student population show participation gaps that mirror the same subgroups in the broader American electorate. Given that similar dynamics appear to be at play, this study of selective incentives points to possible approaches to close the participation gap in elections for public offices.

Second, the election for our experiment was conducted online. The convenience of internet voting reduces the cost of voting, although we were careful in our email to leave it up to the potential voter where to find the voting website and how to perform the task. To ensure similar effects for in-person voting, municipalities and other jurisdictions might have to offer higher payouts than $300 and promote the lottery considerably more. On the other hand, many form of collective action–including registering to vote–take place through online platforms. Our design is directly relevant in such contexts.

Even with these caveats in mind, our findings suggest that new kinds of incentives might engage citizens, particular those with lower socio-economic status, who currently sit on the sidelines during elections. In S1 Fig we provide findings from a survey we conducted among American adults regarding financial rewards for voting. While no method was very popular, non-voters appear significantly more supportive than voters for proposals that provide a financial reward for voting. Since most reforms aimed at improving turnout tend to increase the participation gap between high and low propensity voters [2], the finding that the effects of selective incentives are stronger for lower propensity voters merits additional research. We are not suggesting that a lottery is the appropriate policy solution, but that reformers should think creatively about nudging citizens to the polls by offering some range of benefits rather than simply thinking only of reducing the costs of voting. Previous studies of voluntary acts suggest that offering material rewards has the potential to crowd out pro-social behavior. One classic study of blood donors indicated that monetary compensation undermined the sense of civic duty and willingness to give blood [40]. Panagopoulos [7] in his study using small cash payments to vote did not find evidence of crowding out. However, it is entirely possible that voters forwhom civic duty is a strong motivator might be turned off by getting a material benefit for going to the polls. The challenge is to offer the appropriate incentive for targeted groups in a particular context [41]. The high rates of turnout among eligible U.S. voters during the 19^th^ century was related, in part, to the extrinsic rewards of voting, be it group solidarity, entertainment, drink or cash exchanges [42–44]. Some reforms, such as the secret ballot to prevent vote-buying, seemed entirely justified, even if it did reduce turnout. Others, such as the elimination of entertainment and minor forms of treating seem less so. In 2008, for example, Starbucks had to rescind a widely advertised program to give a free cup of coffee to customers who voted after realizing that they might be violating election laws [45].

Additionally, our experiment may suggest that lotteries may successfully motivate participation in other pro-social activities. Vaccination lotteries have been pursued as a way of dealing with lagging Covid-19 vaccination rates in many states and localities. While results appear mixed [13, 46, 47] we anticipate additional studies in public health to evaluate recent efforts to use lotteries to increase vaccination rates. Lotteries might also be used to encourage individuals to fill out their census forms, register for health insurance, or become organ donors.

Future research should evaluate the mechanisms that motivate people to vote based on incentives. We hypothesized that the potential for a lottery win increased the stakes for participating for those who are typically less interested and engaged. We also suggested that lottery games might trigger a different social network that could motivate different sets of people to engage, particularly those from marginalized groups who may not be embedded in networks where political discussions and participation is as widespread. Based on the design of this study, which did not advertise the lottery broadly, we did not expect social influence to play a significant part in getting students to vote. Additional studies, which could include cities where lotteries are well-advertised, might evaluate the role of social influence through a field experiment.

## Supporting information

S1 Text Full text of emails for experiment. (DOCX)

S1 Table Table showing balanced assignment on sex, race/ethnicity, and first-generation status. (DOCX)

S2 Table Treatment effects for subgroups. (DOCX)

S3 Table Turnout and candidates for UMass student government elections, 2019–2022. (DOCX)

S1 Fig Public opinion on a lottery compared to other financial incentives/penalties. (ZIP)
