# Supplementary material for: A cash lottery increases voter turnout
Source: PLoS One. 2022 Jun 3;17(6):e0268640. doi: 10.1371/journal.pone.0268640 (PMC9165770; doi:10.1371/journal.pone.0268640)
Supplement: S1 Text — (DOCX) [file pone.0268640.s001.docx]

**S1 Text. Full text of emails for experiment.**

Lottery Treatment:

Subject of email: Vote in SGA elections for chance to win $300

Dear (insert name),

Elections are being held this week, Feb 19-21, for representatives to the Student Government Association. As a professor who studies elections, I urge you to vote to choose your leaders, make your voice heard, and participate in the civic life of the [REDACTED] community! Last year only 16% of students voted. ***You have been picked randomly from just 1 in 5 students to participate in a raffle for a chance to win $300 IF YOU VOTE. Five voters will be selected at random and each will win $300****.* You will only be eligible for this reward if you vote. The winnings will be donated through my account at the political science department.

You will receive emails and/or social media providing you with information about candidates and indicating the links where you can vote at the campus site. I encourage you to inform yourself and VOTE!

[Name Redacted], Professor

Department of Political Science

Encouragement Treatment:

Subject of email: Vote in SGA elections

Dear (insert name),

Elections are being held this week, Feb 19-21, for representatives to the Student Government Association. As a professor who studies elections, I urge you to vote to choose your leaders, make your voice heard, and participate in the civic life of the [REDACTED] community! Last year only 16% of students voted.

You will receive emails and/or social media providing you with information about candidates and indicating the links where you can vote at the campus site. I encourage you to inform yourself and VOTE!

[Name REDACTED], Professor

Department of Political Science
