# Supplementary material for: A cash lottery increases voter turnout
Source: PLoS One. 2022 Jun 3;17(6):e0268640. doi: 10.1371/journal.pone.0268640 (PMC9165770; doi:10.1371/journal.pone.0268640)
Supplement: S1 Table — (DOCX) [file pone.0268640.s002.docx]

**S1 Table. Table showing balanced assignment on sex, race/ethnicity, and first-generation status.**

This table presents the distribution of sex, race/ethnicity, and first-generation status among the three groups that we compare in the paper: (a) those who did not receive any email related to this study, (b) those who received only the control (reminder) email, and (c) those who received the treatment (lottery) email. The final column (d) shows the p-value calculation for a difference of proportions test between the latter two groups. We show this calculation since it is the comparison of those two groups that provides our best estimate of the effect of being assigned to a lottery. Note that none of the p-values indicates a statistically significant difference of proportions at the .05 level. While the p-value for Black students is .055, the magnitude of the difference is still quite modest (less than 1 percentage point). Sex and race/ethnicity data is missing for some students. Other lesser populated race/ethnicity categories include American Indian, Hawaiian/Pacific Islander, Multiple, and None.

| Group | (a)  No email | (b)  Control email | (c)  Lottery email | (d)  Test (b) = (c) |
| --- | --- | --- | --- | --- |
| Men | 47.5% | 47.9% | 47.9% | p = 0.979 |
| Women | 45.8% | 45.7% | 46.0% | p = 0.794 |
| Non-Hispanic white | 58.6% | 59.2% | 58.7% | p = 0.659 |
| Hispanic | 6.6% | 7.0% | 6.8% | p = 0.732 |
| Black | 4.2% | 3.9% | 4.8% | p = 0.055 |
| Asian | 15.6% | 15.9% | 15.5% | p = 0.633 |
| Not first-generation | 40.9% | 41.7% | 40.8% | p = 0.427 |
| First-generation | 13.8% | 13.6% | 13.5% | p = 0.899 |
