# Supplementary material for: A cash lottery increases voter turnout
Source: PLoS One. 2022 Jun 3;17(6):e0268640. doi: 10.1371/journal.pone.0268640 (PMC9165770; doi:10.1371/journal.pone.0268640)
Supplement: S2 Table — (DOCX) [file pone.0268640.s003.docx]

**S2 Table. Treatment effects for subgroups**

This tables presents additional subgroup treatment effects that we are able to calculate with the available data. At the top of the table wee show the effects for each of the four largest racial/ethnic groups and in the bottom of the table we show the effects for men and women separately. While the treatment effect estimate for Black students (0.0871) is larger than for Asian students (0.0509), non-Hispanic White students (0.404), and Hispanic students (0.0141), none of these differences are statistically distinguishable from each other at the .05 level. The treatment effects for male and female students are nearly identical. Because our agreement with the University precludes us from receiving individual-level data, we are unable to test these conditional effects simultaneously. All p-values are two-tailed difference of proportions tests, intent-to-treat calculation.

| Group | ATE (Lottery vs. Reminder) | |
| --- | --- | --- |
| Non-Hispanic White students  (N=2,235 & 2,218) | .0404  (p = .001) | |
|  |  | |
| Black students  (N=329 & 228) | .0871  (p =.147) | |
|  |  | |
| Hispanic students  (N=524 & 372) | .0141  (p = .747) | |
|  |  | |
| Asian students  (N=1,185 & 907) | .0509  (p = .049) | |
|  |  | |
| Men  (N=3,619 & 2,474) | .0643  (p <.001) |  |
|  |  |  |
| Women  (N=3,465 & 2,595) | .0626  (p <.001) |  |
