# Supplementary material for: A cash lottery increases voter turnout
Source: PLoS One. 2022 Jun 3;17(6):e0268640. doi: 10.1371/journal.pone.0268640 (PMC9165770; doi:10.1371/journal.pone.0268640)
Supplement: S3 Table — (DOCX) [file pone.0268640.s004.docx]

**S3 Table. Turnout and candidates for UMass student government elections, 2019-2022**

This table puts the 2019 election (in which we fielded our experiment) in context with other recent elections in terms of turnout and competition for offices. As the table shows, the number of candidates competing for various offices is quite similar to other election years. Additionally, turnout in the 2019 election was similar to 2021 but below 2022 (and all are significantly higher than an election held during the pandemic in 2020). Note that the 2020 election was postponed due to the Covid-19 pandemic.

|  | 2019 | 2020* | 2021 | 2022 |
| --- | --- | --- | --- | --- |
| Turnout | 15% | 9% | 14% | 19% |
| Number of Candidates President | 3 | 3 | 3 | 2 |
| Number of Candidates Legislative Seats |  |  |  |  |
| 3rd Year Class (15 Seats) | 13 | 10 | 17 | 11 |
| 2nd Year Class (15 Seats) | 12 | 16 | 12 | 16 |
| 1st Year Class (15 Seats) | 18 | 17 | 22 | 9 |
