# Supplementary material for: A cash lottery increases voter turnout
Source: PLoS One. 2022 Jun 3;17(6):e0268640. doi: 10.1371/journal.pone.0268640 (PMC9165770; doi:10.1371/journal.pone.0268640)
Supplement: S1 Fig — (ZIP) [file pone.0268640.s005.zip › S1_Fig.docx]

In 2012, we included a battery of questions asking 1,000 respondents to the Cooperative Congressional Election Study whether they thought a variety of different proposals for increasing turnout would be a good (or bad) idea. We prefaced our proposals by giving respondents the following statement: “There’s been some discussion about how to improve voter turnout in the United States. Consider the proposals below and indicate whether you think each is a good idea or a bad idea.” Respondents were presented with seven proposals:

1. Every voter would receive $1 for voting
2. Every voter would receive $10 for voting
3. Every person who did not vote would be fined $1
4. Every person who did not vote would be fined $10
5. Every person who did not vote would be fined $100
6. Every voter would be entered in a lottery with one voter winning $220 million
7. Every voter would be entered in a lottery with 100,000 voters winning $2,200

Respondents rated each proposal on a scale from 0 to 100, with 0 indicating that they thought it was a bad idea and 100 indicating that they thought it was a good idea.

Importantly, the 2012 CCES was also matched to records maintained by a voter file firm to determine whether each respondent to the survey had voted in the 2012 election. Among our sample of 1,000 respondents, we were able to confidently identify 529 validated voters and 238 non-voters. We compare the attitudes of voters to those of non-voters since it may be particularly useful to understand which proposals are preferred by individuals who do not currently participate in elections.

The figur presents the average support for each of the proposals among voters and non-voters, along with 95% confidence intervals for those estimates. We use the sampling weights in our calculations to ensure that our sample is representative of American adults. Several clear patterns emerge from this figure. First, none of the proposals receives particularly strong support from respondents. On a scale that ranges from 0 to 100, the most popular proposal only receives an average ranking of about 40 from non-voters (and lower from voters).

A second clear pattern that emerges from the figure is the fact that non-voters appear to be significantly more supportive of proposals that provide a financial reward for voting, compared to voters. Non-voters provide twice as much support for the proposals to reward each voter with either $1 or $100, and they also provide more support for the lottery proposals. Not surprisingly, however, non-voters are no more enthusiastic than voters about proposals to fine those who do not vote.

A third notable pattern from the figure is that overall, and especially among voters, the lottery proposals were the most popular. Indeed, voters provided twice as much support for the lottery proposals as they did for a guaranteed financial reward for voting. Thus, while none of the financial incentive proposals received overwhelming support, the lottery proposals did garner more support than reforms that guaranteed a financial benefit or penalty for voting.
